# Foes or Friends: ABA and Ethylene Interaction under Abiotic Stress

**DOI:** 10.3390/plants10030448

**Published:** 2021-02-27

**Authors:** Maren Müller

**Affiliations:** Department of Evolutionary Biology, Ecology and Environmental Sciences, Faculty of Biology, University of Barcelona, 08028 Barcelona, Spain; maren.muller@ub.edu

**Keywords:** abscisic acid, ethylene, hormone–hormone interaction, abiotic stress, plant hormones, environmental factors

## Abstract

Due to their sessile nature, plants constantly adapt to their environment by modulating various internal plant hormone signals and distributions, as plants perceive environmental changes. Plant hormones include abscisic acid (ABA), auxins, brassinosteroids, cytokinins, ethylene, gibberellins, jasmonates, salicylic acid, and strigolactones, which collectively regulate plant growth, development, metabolism, and defense. Moreover, plant hormone crosstalk coordinates a sophisticated plant hormone network to achieve specific physiological functions, on both a spatial and temporal level. Thus, the study of hormone–hormone interactions is a competitive field of research for deciphering the underlying regulatory mechanisms. Among plant hormones, ABA and ethylene present a fascinating case of interaction. They are commonly recognized to act antagonistically in the control of plant growth, and development, as well as under stress conditions. However, several studies on ABA and ethylene suggest that they can operate in parallel or even interact positively. Here, an overview is provided of the current knowledge on ABA and ethylene interaction, focusing on abiotic stress conditions and a simplified hypothetical model describing stomatal closure / opening, regulated by ABA and ethylene.

## 1. Introduction

ABA and ethylene are two essential plant hormones regulating plant growth, development, and stress responses, including dormancy, vegetative growth, fruits ripening, seed germination, as well as abiotic and biotic stresses [1,2]. Thus, their concentrations are changing constantly under the influence of internal and external signals. ABA and ethylene signaling pathways integrate plant information that leads to the repression or activation of suits of genes, largely by transcription factors (TFs), to modulate plant responses [2,3]. In recent years, the interest to understand these spatial and temporal interactions between ABA and ethylene signaling is growing. However, plant hormone networks are complex and genetic alterations as response to one hormone might cause changes in synthesis, degradation, or sensitivity to other plant hormones. Moreover, specific components in ABA and ethylene pathways might act as nodes to connect multiple pathways/signals of internal or external signals regulating growth, developmental, and stress responses [4,5]. Although mutational analyses in Arabidopsis over the past few decades led to the identification of genes that control ABA and ethylene responsiveness, we are still far from fully understanding the interaction network of ABA and ethylene. It is widely recognized that ABA and ethylene interact antagonistically and influence each other’s synthesis and their signaling transduction pathways. For instance, exogenous ABA application was found to prevent the induction of ethylene biosynthesis [4,6], while ethylene synthesis is increased in ABA-deficient mutants (*Arabidopsis aba2-1* and tomato *flacca*), as compared to the wild-type [7,8,9]. However, ABA and ethylene can not only act antagonistically but they also seem to operate in parallel or interact positively, thus stimulating their biosynthesis or signaling [10,11]. For instance, Luo et al. [10] showed that ABA activates Calcium-dependent Protein Kinase 4 (CPK4) and CPK11 to stabilize 1-Aminocyclopropane-1-Carboxylate Synthase 6 (ACS6), by phosphorylating its C-terminus, and thus promoting ethylene biosynthesis. A recent study [12] suggests that ABA-ethylene signaling is dynamically integrated via protein–protein interaction networks. The character of their relationship, however, appears to be strongly dependent on the endogenous ABA and ethylene levels, which are regulated by the type of tissues, the growth and developmental stage, the plant species, as well as abiotic and biotic stress conditions. Here, a brief overview of both ABA and ethylene biosynthesis and signaling, and its relationship under abiotic stress conditions is provided, a simplified hypothetical model on stomatal movement is proposed, and a perspective on future research is given.

## 2. ABA Biosynthesis and Signaling

ABA belongs to the isoprenoids and its biosynthetic pathways is localized in two places, starting in plastids and ending in the cytosol (reviewed in Vishwakarma et al. [13]). The conversion of β-carotene (C40) to ABA is catalyzed by several enzyme steps. ABA biosynthesis starts with the conversion of zeaxanthin and antheraxanthin to all trans-violaxanthin by zeaxanthin epoxidase (ZEP). All trans-violaxanthin is converted to 9-cis-violaxanthin or 9′-cis-neoxanthin by the 9-cis-epoxy carotenoid dioxygenase (NCED), which leads to the intermediate product xanthoxin. Then, xanthoxin is exported to the cytosol, where it is converted to ABA aldehyde through short-chain alcohol dehydrogenase/reductase (SDR). Finally, ABA aldehyde is oxidized to ABA by ABA oxidase (AAO) [14,15]. ABA is then sensed by ABA receptors (PYrabactin Resistance (PYR)/ Pyrabactin Resistance-Like (PYL) / Regulatory Component of ABA Receptors (RCAR)). In the absence of ABA, however, the negative regulator protein phosphatase 2C (PP2C) associates with Sucrose nonfermenting-1 Related protein Kinase 2 (SnRK2s), thereby, preventing the activation of SnRK2s ([16,17] reviewed in Vishwakarma et al. [13]). In contrast, in the presence of ABA, PYR/PYL/RCAR receptors bind to ABA interacting with PP2Cs, releasing SnRK2s. Then, SnRK2s are activated through the autophosphorylation loop and phosphorylate downstream substrate proteins, such as ABFs (ABA-responsive-element Binding Factor) /AREB (ABA-Responsive Element Binding factors) transcription factors. In addition to nuclear transcription factors, SnRKs are also able to phosphorylate plasma membrane proteins including slow anion channel 1 (SLAC1), potassium channel in Arabidopsis thaliana 1 (KAT1), and NADPH oxidase AtrbohF, which are important for the control of the stomatal aperture [18,19,20,21].

## 3. Ethylene Biosynthesis and Signaling

The biosynthetic pathway of ethylene is a subject of intensive studies and is activated depending on the internal and external stimuli (reviewed in Johnson and Ecker [22], Pattyn et al. [23]). In summary, the ethylene synthesis starts with the formation of S-adenosyl-methionine (S-AdoMet), which is catalyzed by S-adenosyl-L-methionine (SAM) synthetase from the methionine. As the precursor of ethylene, 1-Aminocyclopropane-1-carboxylate (ACC) is the rate-limiting step of ethylene synthesis, due to the conversion of S-AdoMet to ACC, by ACC synthetase. Meanwhile 5′-methylthioadenosine (MTA) is produced as a byproduct generated along with ACC synthesis by ACC synthase, and its recycling back to methionine might serve to maintain a constant concentration of methionine, even when ethylene is rapidly synthesized. On the other hand, malonylation of ACC deprives the ACC pool through the formation of malonyl-ACC (MACC), thus reducing ethylene production. However, ACC oxidase catalyzes the final step of ethylene biosynthesis, using ACC as substrate, with carbon oxide and cyanide formed as byproducts. After its synthesis, ethylene signaling triggers specific biological responses (reviewed in Wang and Ecker [24]; Müller and Munné-Bosch [25]). Five ethylene receptors (Ethylene Response1 (ETR1), ETR2, Ethylene Insensitive4 (EIN4), Ethylene Response Sensor1 (ERS1) and ERS2) localized at the endoplasmic reticulum membrane were identified in Arabidopsis, with ethylene binding occurring at the N-terminal transmembrane domain of the receptors. In the absence of ethylene, ethylene receptors activate a Ralf-like kinase, Constitutive Triple Response1 (CTR1), which in turn negatively regulates the ethylene signaling pathway. However, ethylene binding inactivates receptors and thus deactivates CTR1. These allows EIN2, a positive regulator of ethylene response, to signal downstream to the EIN3/ Ethylene Insensitive-Like Protein1 (EIL1) transcription factors located in the nucleus. Then, EIN3/EIL1 initiates a transcriptional cascade that results in the activation and repression of numerous genes [26]. In Arabidopsis, EIN3 binds to the promoter of Ethylene Response Factor (ERF) genes, activating its transcription in an ethylene-dependent manner, by binding to the specific cis-acting GCC box and DRE elements [27].

## 4. Reciprocity between ABA and Ethylene?

The regulation of stress responses in plants by ABA and ethylene depends largely on the duration of the stress, the developmental stage of the plant, its genetic potential, and the environmental conditions.

**Figure 1:**
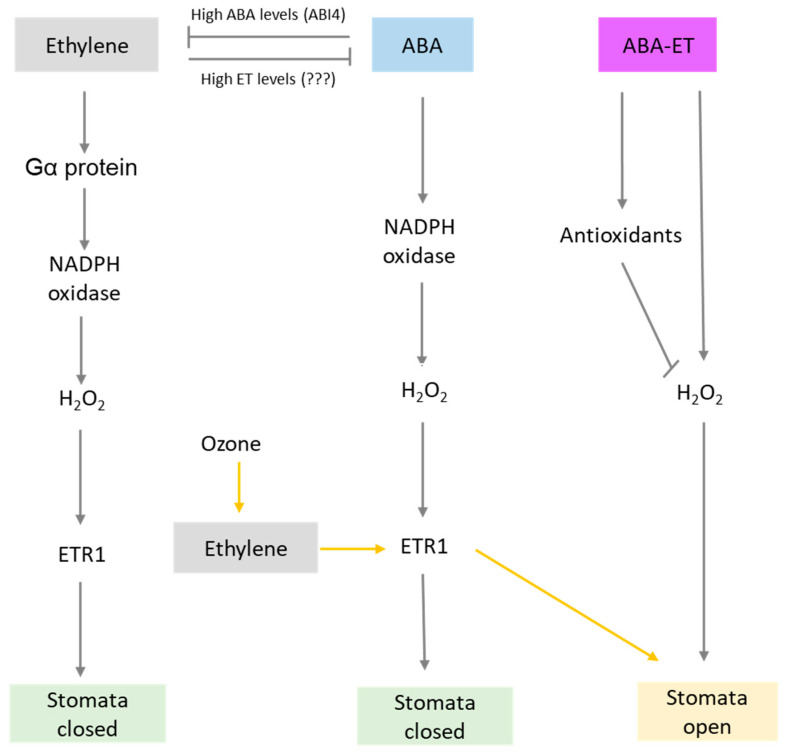
Hypothetical mechanism(s) regulating stomatal closure or opening. ABA and ethylene can individually regulate stomatal closure via H_2_O_2_ and ETR1. While high levels of ABA can inhibit ethylene and vice versa. However, the presence of both ABA and ethylene (ABA–ET) induces antioxidant activity, which in turn reduces H_2_O_2_ contents and thus diminishes stomatal closure (half-open stomata). Under ozone stress, ethylene binds to ERT1, which inhibits the ABA response pathway leading to open stomata (indicated by yellow arrows).

Stomatal closure plays a pivotal role in plant abiotic stress response and is regulated by a complex network of signaling pathways, which leads to an increase stress tolerance (reviewed in Daszkowska-Golec and Szarejko [28]). While ABA is considered to act as the key regulator of stomatal closure under abiotic stress (summarized in Kim et al. [29]; Munemasa et al. [30]; Saradadevi et al. [31]), ethylene is found to act as either a positive or negative regulator [5,32,33,34,35,36,37]. Several studies reported that ABA and ethylene have antagonistic functions in the control of stomatal movement. For instance, in some species (e.g., Arabidopsis, sunflower, tomato), ethylene is reported to induce stomatal opening, thus inhibiting ABA-induced stomatal closure [5,34,38]. Wang and Song [39] proposed that the ethylene receptor ETR1 plays two distinct roles in stomata closure. On the one hand, ERT1 mediates H_2_O_2_ signaling within the ABA response pathway, but acts as receptor for ethylene perception under ozone pollution. Thus, ethylene binding to ERT1 inhibits the alternative signal of the presence of H_2_O_2_, thereby reducing the stomatal response to ABA. However, further research needs to answer whether this ethylene response through ERT1 is limited to ozone only. In addition, a recent study [40] revealed that ERT1 and ERT2 act in both an ethylene-dependent and -independent manner, and thus affect the responses to ABA. H_2_O_2_ is one of the major molecules involved in stomatal movement [41]. Ethylene is reported to regulate stomatal closure, promoting AtrbohF-dependent H_2_O_2_ production through the activation of Gα protein [42]. Exogenous applied ethylene induces H_2_O_2_ synthesis and thus stomatal closure [28,34,41], however, elevated ABA contents, such as that occurring under water stress conditions, negatively modulate ethylene biosynthesis [43]. This might be regulated by the transcription factor ABI4, through the repression of the ethylene biosynthesis genes ACS4 and ACS8 in Arabidopsis [44]. Interestingly, Triticum aestivum plants overexpressing Ethylene Response Factor1 (TaERF1) were highly sensitive to exogenously applied ABA, which resulted in rapid stomatal closure [45]. Moreover, plants overexpressing ERFs (e.g., Jasmonate and Ethylene Response Factor (JERF1), Ethylene Response Factor1 (ERF1), Tomato Ethylene Response Factor (TSRF1)) showed increased ABA contents and enhanced abiotic stress tolerance [27,46,47], indicating that ethylene might regulate ABA biosynthesis through ERF proteins. On the other hand, ABA is found to act as a negative regulator of the ERF1 gene induction [27]. Interestingly, Arabidopsis plants exposed to both ethylene and ABA application resulted in half-open stomata [34]. Nazareno and Hernandez [48] recently constructed a mathematical model of the guard cell transduction network for stomatal aperture. They revealed that stomatal closure under combined ABA-ethylene stimulus is diminished, compared to the effect of individual ABA or ethylene stimulus. The presence of both ABA and ethylene increases the antioxidant activity and reduces H_2_O_2_ contents, thus resulting in diminished closure. However, further research is needed to elucidate whether it is regulated by an ABA-ethylene interaction, or whether they operate in parallel. On the other hand, ABA and ethylene can mutually influence their biosynthetic or signaling pathways, which in turn regulate stomatal movement depending on the environmental conditions (Figure 1; a detailed stomatal regulation is summarized in [28,29,30,31]).

Ethylene is also proposed to be an activator of ABA synthesis under UV-B stress [49]. Ethylene production peaked within the first 5 h and then decreased over the next 24 to 48 h, while ABA contents increased three-fold. However, high ethylene concentration (100 µL/L) blocked ABA accumulation, while exogenous application of ABA (5–5000 µM) inhibited the UV-B-induced ethylene production [49]. In addition to UV-B stress, ethylene also appears to be an activator for ABA production in non-climacteric berry fruits (Vitis vinifera), under non-stress conditions. It was observed that trace endogenous ethylene contents induce ABA production through the transcription of VvNCED1 (VIT_19s0093g00550), which encode a key enzyme in the ABA biosynthetic pathway [50,51]. ABA positively influences ethylene action during tomato (Solanum lycopersicum) fruit ripening by regulating essential genes (e.g., LeACS4, LeACO1) and TFs that regulate ethylene synthesis and sensibility (e.g., MADS-RIN, TAGL1, CNR, and NOR) [52]. Ludwikow et al. [53] revealed that ABI1 regulates ozone-induced ethylene biosynthesis by affecting the ACS6 phosphorylation level. Moreover, a recent study by Li et al. [11] revealed that ABA positively regulated ethylene action under osmotic stress. The authors found that through the ABA-dependent pathway ETR, EIN and ERF in the ethylene signal transduction were all up-regulated.

In conclusion, the ABA-ethylene interaction seems to be strongly dependent on their endogenous levels—(i) high ABA and ethylene concentrations lead to mutual inhibition, (ii) in contrast, low concentrations seem to activate each other’s biosynthesis under certain environmental conditions, (iii) while the presence of both ABA and ethylene stimulates the antioxidant biosynthesis. However, the mechanism of how they repress and specifically stimulate each other at the transcriptional level, or whether they interact or operate in parallel needs further investigation.

## 5. Perspective

In the new era of biological studies, systems biology and quantitative biology, supported omics technologies, and computer simulations are progressively taking on new directions, which will help create a better understanding of hormone–hormone interactions in plants. The studies of ABA and ethylene and in particular their co-actions will shed new light on the molecular mechanisms. Moreover, the understanding of the ABA and ethylene interaction network can be further exploited to produce transgenic plants with improved stress tolerance without loss of yield, and thus to be able to withstand adverse climatic conditions. The fine-tuning of CO_2_ assimilation and transpiration will play a crucial role in this. However, many efforts are still required to uncover in detail how ABA and ethylene interaction regulates the stress signal transduction pathways. The role of ethylene in guard cells and its interaction with ABA is still not fully understood. The mechanisms through which stress upregulate ABA and ethylene biosynthesis genes need further investigation. For instance, stomata often fail to close under chilling stress conditions and stomatal responsiveness to exogenously applied ABA is reduced [54]. In addition to ABA and ethylene interaction, the molecular mechanisms of interaction with other plant hormone signaling pathways remain to be largely elucidated. While many questions remain open, research should not ignore paving the way to address the molecular events underlying stress responses even more in other plant species than Arabidopsis, with the prospect of improving stress tolerance performance of crop plants.
